# Potential Markers of Progression in Idiopathic Parkinson’s Disease Derived From Assessment of Circular Gait With a Single Body-Fixed-Sensor: A 5 Year Longitudinal Study

**DOI:** 10.3389/fnhum.2019.00059

**Published:** 2019-02-19

**Authors:** M. Encarna Micó-Amigo, Idsart Kingma, Sebastian Heinzel, Sietse M. Rispens, Tanja Heger, Susanne Nussbaum, Rob C. van Lummel, Daniela Berg, Walter Maetzler, Jaap H. van Dieën

**Affiliations:** ^1^Department of Human Movement Sciences, Vrije Universiteit Amsterdam, Amsterdam Movement Sciences, Amsterdam, Netherlands; ^2^Department of Neurology, Christian-Albrechts-University, Kiel, Germany; ^3^Personal Health Department, Philips Research Europe, Eindhoven, Netherlands; ^4^Department of Neurodegeneration, Center of Neurology, Hertie Institute for Clinical Brain Research, University of Tübingen, Tübingen, Germany; ^5^DZNE, German Center for Neurodegenerative Diseases, Tübingen, Germany; ^6^McRoberts B.V., The Hague, Netherlands

**Keywords:** walking, accelerometry, movement disorders, gait analysis, Parkinson’s disease

## Abstract

**Background and Aim:** Development of objective, reliable and easy-to-use methods to obtain progression markers of Parkinson’s disease (PD) is required to evaluate interventions and to advance research in PD. This study aimed to provide quantitative markers of progression in idiopathic PD from the assessment of circular gait (walking in circles) with a single body-fixed inertial sensor placed on the lower back.

**Methods:** The assessments were performed every 6 months over a (up to) 5 years period for 22 patients in early-stage PD, 27 patients in middle-stage PD and 25 healthy controls (HC). Longitudinal changes of 24 gait features extracted from accelerometry were compared between PD groups and HCs with generalized estimating equations (GEE) analysis, accounting for gait speed, age and levodopa medication state confounders when required.

**Results:** Five gait features indicated progressive worsening in early stages of PD: number of steps, total duration and harmonic ratios calculated from vertical (VT), medio-lateral (ML), and anterior-posterior (AP) accelerations. For middle stages of PD, three gait features were identified as potential progression markers: stride time variability, and stride regularity from VT and AP acceleration.

**Conclusion:** Faster progressive worsening of gait features in early and middle stages of PD relative to healthy controls over 5 years confirmed the potential of accelerometry-based assessments as quantitative progression markers in early and middle stages of the disease. The difference in significant parameters between both PD groups suggests that distinct domains of gait deteriorate in these PD stages. We conclude that instrumented circular walking assessment is a practical and useful tool in the assessment of PD progression that may have relevant potential to be implemented in clinical trials and even clinical routine, particularly in a developing digital era.

## Introduction

Parkinson’s disease (PD) is a neurological disorder characterized by progressive impairments of balance, gait and mobility. These symptoms often cause disability and lead to the loss of independence, which reduce the patient’s physical activity (Speelman et al., 2011) and quality of life (Jankovic, 2008). Moreover, gait deficits could lead to multisystem deconditioning (Speelman et al., 2011) and recurrent falls (Wenning et al., 1999), and these to injury and reduced survival (Wenning et al., 1999; Speelman et al., 2011). The aetiology of idiopathic PD is unidentified and there is no known cure. Neuroprotective or even neuromodulator therapies to stop neurodegeneration are still lacking, although there is increasing evidence for therapeutic strategies that may delay disease progression (Sarkar et al., 2016).

At present, expert opinion supported by subjective and low-sensitive tools, such as semi-quantitative and qualitative clinical assessments, is considered the gold standard in patient assessment (Evans et al., 2011). Clearly, the evaluation of therapeutic interventions, as well as the understanding of the relation between symptoms and neurodegeneration would benefit from the presence of objective markers sensitive to changes in the long (Holford and Nutt, 2008) and short term. Moreover, such markers are needed for a more precise and earlier detection of the disease and might add value in the characterization of high-risk populations, which is essential for a better understanding of PD aetiology (Chen et al., 2013). Thus, the development of objective, reasonably priced, reliable and easy-to-use methods to obtain markers of the disease is required.

With this aim, urinary dysfunction has been investigated and was found to be an early marker of PD progression, but its usability is limited by the fact that only 30–65% of patients with PD present this particular dysfunction (Picillo et al., 2017). Furthermore, digitomotography (Maetzler et al., 2015) and pegboard (Haaxma et al., 2010) performance assessments have been shown to be reliable methods to identify motor dysfunction of the upper-extremities in early stages of PD. Recently, longitudinal (over a 4 years span) differences in deterioration of pegboard performance between patients in early stages of PD and HC have been shown (Heinzel et al., 2017). Nevertheless, their potential as a progression marker requires further validation.

Methods based on quantitative assessments of gait have been proven to successfully identify subtle motor impairments in early to middle stages of PD (Barth et al., 2011; Micó-Amigo et al., 2017). In this context, body-fixed-sensors (BFS; commonly including accelerometers, gyroscopes and magnetometers) may have an important role, particularly due to their portability, low cost and good usability (Godfrey et al., 2015; Del Din et al., 2016a,b; Morris et al., 2017). Their use may enhance objective, sensitive and reliable clinical testing (Maetzler and Hausdorff, 2012; Espay et al., 2016). However, progression markers obtained from motor assessments with BFS still require further development.

Turning impairments seem to be independent from disturbances of linear walking (Guglielmetti et al., 2009). Particularly, the turning while walking section of the timed up and go test (a sequence of sit-to-stand, walking, turning while walking, and stand-to-sit tasks) has been identified as the most sensitive to PD gait impairments (Palmerini et al., 2013). Moreover, neural processes related to turning might be more susceptible to functional impairments associated with PD when compared to linear walking tasks (Crenna et al., 2007). Interestingly, the PD-associated neurodegeneration of the basal ganglia likely degrades the performance of turning while walking (Wichmann et al., 2001). Difficulties with turning while walking are common in patients with PD, possibly due to impairment of inter-limb synchronization (Crenna et al., 2007), deficits in balance control (Mellone et al., 2016) or loss of axial flexibility (Visser et al., 2007). Moreover, limitations in turning are among the early motor deficiencies of PD (Visser et al., 2007) and contribute to restricted mobility, falls and a reduced quality of life (El-Gohary et al., 2013).

From a clinical point of view, axial and gait symptoms develop faster than other motor symptoms of PD (Evans et al., 2011). Thus, objective and quantitative assessment of turning in gait seems promising for the study of PD progression. Therefore, the aim of this prospective study was to identify objective markers of progressive motor deficits in idiopathic PD from the assessment of continuous turning while walking with a single BFS placed on the lower back.

## Materials and Methods

### Participants

As part of the prospective observational MODEP study (Modeling epidemiological data to study PD progression), bi-annual assessments were performed every 6 months over a 5-years period (up to 10 visits) in 74 participants, 49 patients diagnosed with idiopathic PD and 25 healthy controls (HC). All participants were recruited from the outpatient clinic of the Department of Neurodegeneration, Center of Neurology, University Hospital of Tübingen, Germany. In a previous publication, fine motor skills were assessed as potential markers of PD progression, in a partially overlapping study population (Heinzel et al., 2017). For the present study, which focused on gait instead of fine motor skills, additional subjects (*n* = 10) were included, and follow-up assessments performed over 5 instead of 4 years were used.

The Declaration of Helsinki was respected; local ethic committee approval was obtained (Medical Faculty, University Hospital of Tübingen, No. 46/2010 BO1) and all subjects provided informed written consent for participation in the study and for publication of individual, anonymized data. The participants were selected according to the following inclusion criteria: (a) age between 40 and 85 years; (b) stable medication for 2 weeks prior to inclusion; (c) absence of cognitive impairment based on a minimum score of 25 points in the Mini Mental State Examination (MMSE) (Folstein et al., 1975). All participants underwent a clinical assessment that included medical history, medication intake and neurological examination. The participants of the PD group were diagnosed with idiopathic PD according to the United Kingdom Brain Bank Society criteria (Hughes et al., 1992) and did not present any other neurological disorder, nor dysexecutive syndrome. The participants of the control group had no neurological disease. A detailed description is also available in our previous publication (Heinzel et al., 2017).

Considering that the rate of change of motor symptoms is different between early and mid-advanced stages of PD (Kieburtz et al., 1996; Fahn et al., 2004; Maetzler et al., 2009), *a priori* classification of the PD group was performed. Thus, each group was compared to the reference group (HC) in order to study motor symptom progression at different stages of the disease. Analogous to previous work (Heinzel et al., 2017), patients were stratified into early-stage PD (Early-PD; < 4 years, *N* = 22) and middle-stage PD (Mid-PD; ≥ 4 years, *N* = 27) based on the duration after clinical PD diagnosis. An overview of demographic and clinical data is presented in Table 1. For the Early-PD, most of the participants were diagnosed with PD within less than 3 years before baseline: 5 patients (22.7% of the Early-PD group) were diagnosed within less than 1 year before baseline assessment, 9 patients (40.9% of the Early-PD group) were diagnosed within less than 2 years and more than 1 year before baseline assessment, 7 patients (31.8% of the Early-PD group) were diagnosed within less than 3 years and more than 2 years before baseline assessment and only 1 patient (13.6% of the Early-PD) was diagnosed within less than 3 years and more than 2 years before baseline assessment.

**Table 1:** Demographics and clinical data of participants at baseline (first visit).

		HC (*N* = 25)	PD-early (*N* = 22)	PD-mid (*N* = 27)	HC–PD-early *p*-values	HC–PD-mid *p*-values	PD-Early – PD-mid *p*-values
Demographics	Gender (female)	10 (40.0%)	9 (40.9%)	10 (37.0%)	0.92	0.84	0.79
	Age [years]	63.6 [50–75]	61.2 [41–73]	65.3 [43–76]	0.27	0.30	0.06
	Total height [m]	1.71 ± 0.08	1.74 ± 0.10	1.73 ± 0.08	0.33	0.41	0.79
	Mass [kg]	76.0 ± 11.3	81.0 ± 19.1	78.5 ± 13.0	0.27	0.47	0.59
	BMI [kg/m^2^]	25.9 ± 3.2	26.7 ± 5.2	26.2 ± 3.7	0.54	0.79	0.69
	MMSE (1–30)	28.9 [26–30]	28.3 [26–30]	28.4 [25–30]	0.11	0.12	0.70
	Academic Education [years]	11.0 [9–13]	10.5 [9–13]	11.0 [9–13]	0.38	0.89	0.30
	Disease duration [years since diagnose]		1.2 [0–3]	7.1 [4–11]			< 0.001
	Disease duration [years since first symptoms]		2.0 [0–4]	8.0 [5–11]			< 0.001
	Age at diagnose [years]		60.0 [40–72]	58.2 [35–71]			0.26
	Age at manifestation [years]		59.2 [39–70]	57.3 [35–71]			0.22
Clinical	Hoehn and Yahr (0–4)		1.7 [1–3]	2.4 [1–4]			< 0.001
	Number of PIGD–Number of TD		7–12	15–12			
	MDS–UPDRS III (0–132)	1 [0–7]	21.5 [5–32]	35.8 [8–68]	< 0.001	<0.001	<0.001
	Tremor subscore of UPDRS III (0–44)		24.8 ± 8.9	40.9 ± 14.1			<0.001
	Gait subscore of UPDRS III (0–20)	0.0 [0.0–1.0]	1.1 [0–3]	3.7 [0–14]	< 0.001	< 0.001	< 0.001
	Daily levodopa medication equivalent dose [mg]		229.7 [0–607]	689.3 [80–1300]			< 0.001


**Data is presented as mean ± standard deviation for the parametric data and mean [range] for the non-parametric data. In case of gender, data is presented as a number of females and (the percentage of females over the total number of participants for each group).* P-values for comparisons between groups are indicated. Abbreviations: Early-PD, patients at early stages of Parkinson’s disease; Mid-PD, patients at middle stages of Parkinson’s Disease; HC, healthy controls; BMI, body-mass-index; MMSE, Mini-Mental-State-Examination score; MDS – UPDRS III, motor section of Unified Parkinson’s disease rating scale as defined by the Movement Disorders Society; PIGD, patients with predominant postural instability and gait disorder clinical phenotype; TD, patients with tremor dominant clinical (Stebbins et al., 2013). Levadopa equivalent dose was calculated as recommend by Tomlinson et al. (2010).*

Visits differed in medication state within and between-subjects (16.4% of the assessments were in ON medication state; Elshehabi et al., 2016). The condition ON medication was defined as a time period of 30 minutes to 3 hours after the intake of the usual dose of dopaminergic medication (prescribed by the neurologist for an optimal medical treatment) and considering each participant’s perception of having a “Good On Phase.” Dyskinesia induced by treatment was uncommon in the PD group (six subjects presented dyskinesia at a single visit, one subject at two visits and three subjects at three visits). These patients presented dyskinetic movements after 7.9 years (on average) from the clinical diagnosis. At baseline, twelve patients presented motor fluctuations induced by medication, eleven of them in middle-stage and one in early-stage PD. Six patients in middle-stage PD presented freezing of gait symptom. Four participants were rated with a score of 1 out of 4 in the freezing of gait section of the Unified Parkinson’s Disease Rating Scale (UPDRS) (Stebbins et al., 2013), while two participants were rated with a score of 3 out of 4.

### Protocol

All subjects wore their own shoes and a BFS on the lower back (see below), while they continuously walked three rounds around a circle of 1.2 m diameter at their preferred speed. This protocol was performed twice at each visit, once in clock-wise and once in counter-clock-wise direction. After a verbal countdown the subjects started to walk around the circle. The trial ended after the subjects completed the third round and reached their original position. The examiner stood always on the cloth (circle) and turned with the participants while extending the arms, so that the examiner’s front of the body was in the direction of the participant and potentially able to help him/her in case of postural instabilities. Thus, the dimension of the diameter (1.2 m) was chosen to permit the examiner to stand/turn with the participant at an appropriate distance to safeguard the participant. Moreover, turning around an inner diameter of 1.2 m approximates turning behaviors that may occur in several daily-relevant situations, characterized by reduced indoors (in the kitchen, living room, supermarket, etc.) and outdoors (in the bus, on the sidewalk of the street, etc.) spaces.

### Instrumentation

**FIGURE 1:**
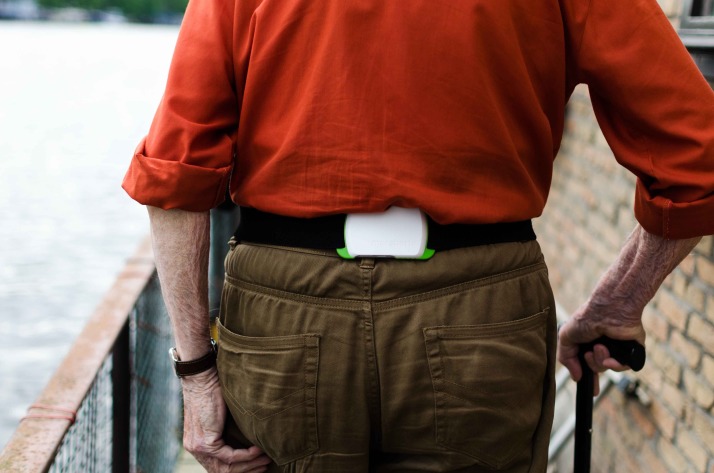
Sensor positioned on the low-back (L5).

The measurement system consisted of a BFS (DynaPort^®^ Hybrid, McRoberts), a remote control and a portable computer on which the DynaPort McRoberts software was installed. The sensor consists of a triaxial accelerometer and a triaxial gyroscope, and stores data at a rate of 100 samples per second. The accelerometer is a DC type sensor and therefore it is also sensitive to gravity. It has a range of ± 19.62 m/s^2^ and a resolution of 0.00981 m/s^2^. The sensor was inserted in an elastic belt, placed around the waist so that the sensor was positioned at the level of the lowest lumbar (L5) vertebra (Figure 1). Assessments were performed under clinical supervision to carefully control sensor placement and orientation. All tests were implemented on a computer. Dedicated software was activated with a remote control to initiate and stop data collection.

### Pre-processing of Data: Segmentation in Step Cycles and Realignment

Acceleration signals were first aligned to correct for potential static sensor misalignments (Moe-Nilssen, 1998; Tundo et al., 2013). Subsequently, the mean value over the first 20 samples (during the standing initial phase) was subtracted from each signal (thereby also removing the offset in the VT acceleration signal caused by the effect of gravity). With the aim to automatically segment the signals into step cycles, a template-matching method was applied to the low-back AP acceleration signal (Micó-Amigo et al., 2016, 2017). The performance of this algorithm has been validated previously for 5 m straight walking (Micó-Amigo et al., 2016, 2017).

#### Validation of Segmentation in Step Cycles

In a separate study, the performance of the algorithm was tested for circular gait in a data set referred to as “Validation cohort”. This data set, for which straight walking data have been published previously (Micó-Amigo et al., 2016), comprised 14 subjects with idiopathic PD (23.1% women, H&Y: 2–3, average age: 61.5 ± 8.9 years and with a minimum score of 25 points in the MMSE) and 13 healthy control subjects (28.6% women, average age: 62.4 ± 10.0 years). The participants were assessed at the University Hospital of Tübingen and at the Vrije Universiteit Amsterdam after local ethic committee approval (Tübingen 140622 and METC VUmc: 2010/290, respectively). All subjects provided informed written consent for participation in the study and for publication of individual, anonymized data.

Similar to our previous work, based on short straight gait trials (Micó-Amigo et al., 2016, 2017), the performance of the algorithm was tested by evaluating the absolute average difference in stride durations between estimates obtained from the application of the algorithm to low-back accelerometry and to heel accelerometry. Heel accelerometry was only used in the “Validation cohort” and not in the longitudinal study.

### Calculation of Gait-Parameters

A comprehensive set of twenty-four gait characteristics was estimated from triaxial acceleration signals. Raw unfiltered data were analyzed to assure that no information was lost or altered due to filtering (Bisi et al., 2014). Most of the calculated characteristics have been shown to distinguish between gait patterns of persons with PD and controls, and between fallers and non-fallers (Weiss et al., 2011; Rispens et al., 2014b).

The following features were obtained:

- **Number of steps**, calculated as the total number of cycles detected with the step segmentation algorithm.
- **Total duration**, calculated as the time interval between the start and end of the trial, which was manually marked based on visual inspection of signals.
- **Step time asymmetry**, calculated as the absolute difference between the mean duration of the steps performed with the right leg and with the left leg. Larger values of this feature reflect higher gait asymmetry.
- **Median stride time**, calculated as the median duration of two accumulated steps (obtained from the segmentation algorithm), i.e., complete gait cycles.
- **Stride time variability**, calculated as the standard deviation of stride durations, excluding the highest and lowest 5%.
- **Signal standard deviation (SD)**, estimated for each signal as its variability around the mean (for each of the three directions of accelerometry). This feature was referred to as “movement intensity” in previous studies (Menz et al., 2003a; Hees et al., 2009).
- **Step regularity**, estimated for vertical (VT) and antero-posterior (AP) accelerations as the normalized unbiased auto-covariance for a lag of one step time (Moe-Nilssen and Helbostad, 2004). This features thus reflects the similarity between subsequent steps of the acceleration pattern over a step. Values of this feature close to 1.0 (maximum possible value) reflect repeatable patterns between subsequent steps.
- **Stride regularity**, estimated for VT and AP accelerations as the normalized unbiased auto-covariance for a lag of one stride time (Moe-Nilssen and Helbostad, 2004). Values of this feature close to 1.0 reflect repeatable patterns between strides.

The following features are based on the spectral content of signals. We calculated the power spectral density with the function *pwelch* from the Matlab signal processing toolbox. For that, we used a Hanning window of 900 samples (of slightly shorter duration than the shortest performed gait trial), with 50% window overlap and 9000 Nfft (number of fast Fourier transform coefficients for multi-taper spectrum).

- **Harmonic ratios**, estimated for each direction as described by Menz et al. (2003a). Harmonic ratios of acceleration signals in VT and AP directions were calculated as the sum of even harmonics divided by the sum of odd harmonics, since these signals have two phases per stride. Harmonic ratios from ML acceleration were calculated as the sum of odd harmonics divided by the sum of even harmonics, since acceleration signals in mediolateral (ML) direction are monophasic per stride. This measurement reflects the rhythmicity of periodic patterns and relates to gait symmetry (Bellanca et al., 2013; Pasciuto et al., 2017). Thus, higher values of this feature are related to more rhythmic, paced and symmetric gait patterns.

- **Index of harmonicity**, estimated for each direction as the power spectral density (PSD) of the fundamental frequency (first harmonic) divided by the cumulative sum of the power spectral density of the first six harmonics (Lamoth et al., 2002; Rispens et al., 2014a). This measure, proposed by Lamoth et al. (2002), reflects gait smoothness. Thus, values approaching the maximum value of 1.0 indicate a smoother gait pattern, which may reflect a less vigorous/more cautious movement pattern, whereas smaller values might indicate more erratic movements.

**- Normalized peak power**, estimated from each detrended acceleration signal (VT, ML, AP) as the magnitude of the PSD at the dominant peak, normalized by the total integrated PSD. This feature represents the relative strength of the signal at the most dominant frequency and reflects the periodicity of the signal (Weiss et al., 2011). Larger values of this feature indicate a more periodic gait pattern.

**- Width of peak power**, estimated from each detrended acceleration signal (VT, ML, AP) as the width of the dominant peak of the PSD. This feature is a measure of frequency dispersion and is related to the variability of the dominant cycles of the signal (step cycles in VT and AP, stride cycles in ML). Larger values of this feature indicate a less consistent gait pattern (Weiss et al., 2011).

To check for lateralization effects, we did separately analyze clockwise and counter-clockwise trials. In addition, we analyzed a selection of trials such that for the PD patients the side most impaired by PD was on the inside of the circle or on the outside of the circle. As this showed no relevant effects of lateralization (see Discussion and Supplementary Appendix B), the average of gait features extracted from both trials (clock-wise and counter-clock-wise direction) was obtained and used for further analysis.

### Potential Markers of Progression in Parkinson’s Disease

To identify progression markers in different stages of PD we consider several criteria, which are all required to be met. For markers of early-stage PD, these features should show:

E1) a Group (Early-PD vs. HC) x Time interaction in the expected direction, reflecting faster annual decline of gait quality in the Early-PD compared to HCs.
E2) a significant percentage of annual change in the Early-PD group, in the expected direction.
E3) an association to the summed score of the items concerning gait assessment from the motor section of the Unified Parkinson’s Disease Rating Scale (Stebbins et al., 2013), referred to as gait-UPDRS III.
E4) a significant effect of Group (Mid-PD vs. HC) in the expected direction, reflecting impaired gait performance of the Mid-PD with respect to HCs.

Criterion E3 was taken into account because clinical measures are currently still considered gold standards for the assessment of progression in PD (Evans et al., 2011). The fourth criterion (E4) implies that the gait symptoms that worsen (i.e., their severity increases with time) in early stages PD are expected to still be present at more advanced stages of the disease, such as middle-stages PD. Thus, these gait symptoms remain at baseline in middle stages of the disease. Notice that criterion (E4) does not need to show a change over time in Mid-PD (ceiling may have been reached), but it should solely be different from HC in Mid-PD at baseline.

For markers of middle-stage PD, all the criteria required to be met are:

M1) a significant Group (Mid-PD vs. HC) x Time interaction in the expected direction, reflecting faster annual decline of gait quality in the Mid-PD compared to the HC.
M2) a significant percentage of annual change in the Mid-PD group in the expected direction.
M3) an association with the gait-UPDRS III score in the expected direction.

Since no data on advanced stages of PD were available, no criterion analogous to criterion E4 could be defined for the Mid-PD group.

### Statistical Analysis

All calculations were performed with a custom Matlab program (Natwick, Massachusetts: The MathWorks Inc., R2016a). The Shapiro-Wilk test and Shapiro-Francia test (for platykurtic and leptokurtic distributions, respectively) were used to test normality of data distribution of demographic and clinical data. Accordingly, unpaired *t*-tests and Wilcoxon Rank tests were used to assess differences between groups for the demographic and clinical data. The level of significance was set to α = 0.05 (two-sided).

To reduce variance caused by day-to-day differences as well as measurement errors, gait features and clinical scores assessed from biannual visits were averaged to obtain annual data, i.e., year 1 (baseline) represents the average of visit 1 and visit 2 data, year 2 (visits 3–4), year 3 (visits 5–6), year 4 (visits 7–8) and year 5 (visits 9–10). In case of single missed visits, the remaining value of that year was taken as the average.

All features were statistically analyzed with generalized estimating equations (GEE) (Shults and Ratcliffe, 2008), using identity-link functions with normal distributions. GEE handles missing data by making assumptions on the mean and variance-covariance structure of the outcomes. Moreover, GEE yields consistent estimators, provided that the model for the marginal means of the outcomes is correctly specified (Paik, 1997; Wang and Paik, 2011). Logarithmic transformation was applied to improve distribution of skewed data (Keene, 1995). In case of negative data, an offset value was added to guarantee positive data prior to logarithmic transformation. In addition, all gait features were scaled to obtain zero means and unit variances (z-score transformation) (Milligan and Cooper, 1988), to allow easy comparison of model coefficients between the proposed variables. We used the between-subject variance over the combined groups for this normalization. Inspection of correlations between measurement points confirmed the assumption of an equicorrelated structure of the data. The models comprised the factors Group (coded with 0 = HC and 1 = Early-PD or Mid-PD in their respective model), Time (number of years, with baseline set to 0) and the Group x Time interaction. Time, expressed in years from 0 to 4, was used as a numerical variable here. Hence the regression coefficient reflects the annual change. In addition, the effect of potential confounders was analyzed by including the following factors: total circular walking duration (to assess the effect of gait speed), age and ON/OFF medication status (0 = OFF, 1 = ON, 16% of all visits). The HC did not take any anti-parkinsonian medication, therefore, for this group the score was always 0. After including all potential confounders (for each analysis), we identified the confounder with the highest *p*-value and this was removed if not significant (with α < 0.05). Subsequently, the analysis was repeated with the remaining factors until only significant confounders were kept in the analysis. In the case of GEE analyses of clinical ratings and the feature total duration of trial, only age and ON/OFF medication status were evaluated as potential confounders. For reference, the same analysis was performed for the gait-UPDRS III scores.

Criteria E1 and M1 were assessed based on the model coefficients of the Group × Time interactions. Criterion E4 was assessed based on the model coefficient for Group in the analysis of the Mid-PD vs. HC. To assess criteria E2 and M2, we calculated the percentage of annual change with respect to the baseline. This was calculated separately for each group by applying GEE analysis on non-transformed data, including the factor Time and the significant confounders identified in the preceding analyses. The resulting model coefficients and standard errors of the Time factor were divided by the mean value of the data obtained in the first year of all subjects within the group. The assessment of criteria E3 and M3 was based on a GEE analysis, performed to determine the regression of each gait feature on gait-UPDRS III. Data were transformed and normalized (log transformation, z-score transformation). The following potential confounders were considered: total duration, age and medication status, while only significant confounders were kept in the analyses.

In view of the use of multiple criteria, we accepted single criteria to be met with α = 0.10. Assuming independence of criteria, this implies α-levels of 0.0001 (0.1^∧^4) for the definition of progression markers in the Early-PD and 0.001 (0.1^∧^3) for the Mid-PD. These α-levels are conservative compared to Bonferroni correction, which for the 24 gait features would yield an α-level of 0.002. We additionally report differences between Early-PD and Mid-PD vs. HC, for which we used a conventional α-level of 0.05.

## Results

### Descriptive Statistics

Descriptive statistics of clinical parameters for all the groups are presented in Table 1. Demographic variables did not significantly differ between the HC and the Early-PD groups, nor between the HC and the Mid-PD groups. The patient visits per year of follow-up were: 100% for year 1 (74 participants), 82.3% for year 2 (61 participants), 77.0% for year 3 (57 participants), 63.5% for year 4 (47 participants) and 51.4% for year 5 (38 participants). An overview of data availability in the MODEP cohort is presented in Supplementary Appendix A, Table A1.

### Signal Segmentation Into Step Cycles

Based on visual inspection, the step detection algorithm applied to low-back accelerometry detected all the steps/strides without false positives and without false negatives when compared to heel accelerometry in our separate validation sample. In HC, absolute average differences between low-back and heel accelerometry in stride duration were: 20.7 ± 10.0 ms (3.5 ± 1.5% of average stride duration), with an ICC = 0.97. For the PD group, these differences were: 22.1 ± 8.9 ms (3.6 ± 1.4% of average stride duration), with an ICC = 0.92. No significant differences were found in estimation errors between the HC and the PD groups (*p* = 0.48). The magnitude of the differences obtained was comparable to findings in our previous study (Micó-Amigo et al., 2016), which segmented short trials of straight gait in step cycles with acceptable accuracy.

### Progression Markers of Early-Stage PD

**FIGURE 2:**
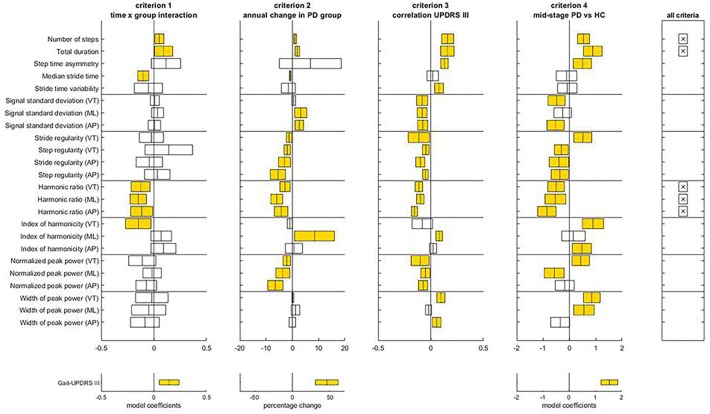
Assessment of the criteria to identify progression makers in Early-PD. For each gait feature, means and confidence intervals of model coefficients for the interaction of time and group, for the percentage of annual change in the Early-PD, for the model coefficient of the regression to gait-UPDRS III scores, and for the model coefficient for group in the Mid-PD vs HC comparison are presented. The right panel indicates which features satisfy all four criteria. The lower panels provide results for the gait-UPDRS III scores as a reference. Note the difference in x-axis scale for percentage annual changes of gait features and gait-UPDRS III scores.

Figure 2 illustrates the assessment of gait features against the four criteria for progression markers of early-stage PD. The complete statistical results underlying this assessment can be found in Supplementary Appendix A (Tables A3, A5). Five gait features met all four criteria: number of steps, total duration and harmonic ratio of accelerations in the three axes (VT, ML, and AP). Notice that gait-UPDRS III presented larger model coefficients and annual change than any of the gait features.

With respect to criteria E1 and E2, Time x Group interactions were found for seven gait features, all in the expected direction. For these features, absolute model coefficients ranged from 0.05 to 0.15. The **number of steps** increased slightly faster in the Early-PD than in the reference group; the annual change of non-transformed data relative to the first year was 1.03 ± 0.33% for the Early-PD. **Total duration** reflected a faster decline of gait speed for the Early-PD (1.91 ± 0.17% annual change) than the HC. **Median stride time** declined faster in the Early-PD relative to the HC, with an annual change in non-transformed data for the Early-PD of -0.85 ± 0.18%. **Harmonic ratios (VT, ML, and AP)** presented a faster decrease in the Early-PD with respect to the HC, with annual changes for the Early-PD group of -2.79 ± 1.21% (VT), -5.93 ± 1.38% (ML), and -4.27 ± 1.58% (AP). For gait-UPDRS III scores the interaction model coefficient was 0.14, with a significant percentage of annual change of 46.09 ± 9.24%. With respect to criterion E3, gait-UPDRS III scores were significantly associated with all the parameters that satisfied the previous criteria, except for median stride time. Finally, with respect to criterion E4, all five parameters that satisfied the first two criteria were different between the Mid-PD vs. HC groups and the direction of these differences was consistent with gait impairment in the PD group.

### Progression Markers of Middle-Stage PD

**FIGURE 3:**
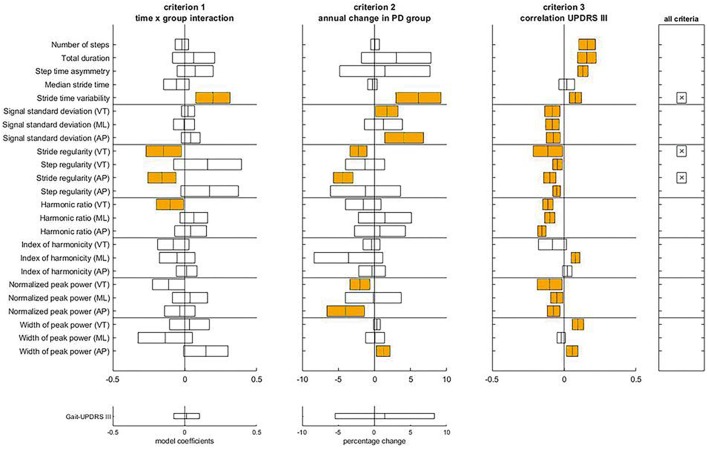
Assessment of the criteria to identify progression makers in Mid-PD. For each gait feature, means and confidence intervals of model coefficients for the interaction of time and group, for the percentage of annual change in the Mid-PD, and for the model coefficient of the regression to gait-UPDRS III scores are presented. The right panel indicates which features satisfy all three criteria. The lower panels provide results for the gait-UPDRS III scores as a reference.

Figure 3 illustrates the assessment of the gait features against the three criteria for progression markers of middle-stage PD. The complete statistical results underlying this assessment can be found in Supplementary Appendix A (Tables A4, A5). For the Mid-PD group, three gait features met all three criteria: stride time variability, and stride regularities from VT and AP acceleration. In contrast to the Early-PD, gait-UPDRS III did not show a significant change over time in the Mid-PD, nor a difference in time course between Mid-PD and HC groups.

With respect to criteria M1 and M2, differences in progression between Mid-PD and HC groups were observed for **stride time variability** and **stride regularity VT and AP**. Specifically, Mid-PD patients increased stride time variability more rapidly than the HC; annual changes of stride time variability of non-transformed data were non-significant for the HC, and significant at 6.09 ± 1.90% for the Mid-PD. In line with this, also stride regularity showed a progressive decrease in the Mid-PD, with negative annual changes of stride regularity VT and AP only in the Mid-PD: -2.21 ± 0.71% and -4.34 ± 0.82%, respectively. With respect to criterion M3, gait-UPDRS III scores were significantly associated with all the parameters that satisfied the other two criteria.

### Baseline Differences Between Healthy Controls and Patients in Early and Middle Stages of PD

(Table A2) Supplementary Appendix A presents mean and standard deviation values within groups of each gait feature (non-transformed and non z-score normalized) at baseline (year 1). Nine gait features were significantly different between HC and Early-PD groups (Figure 1 and Supplementary Appendix A, Table A3. Absolute model coefficients ranged from 0.39 to 0.92, with three of them above 0.7. As expected, the Early-PD performed the protocol slower (longer duration) than the HC. Moreover, the Early-PD showed a lower step and stride regularity in AP, lower harmonic ratio AP, higher index of harmonicity VT, lower normalized peak power AP and a higher width of peak power in the three directions (VT, ML and AP). Gait-UPDRS III scores showed the largest beta value for the group effect in the comparison Early-PD vs. HC.

When comparing the Mid-PD vs. the HC, fifteen gait features were different between groups, with model coefficients ranging from 0.30 to 0.89 and four above 0.7 (Supplementary Appendix A, Table A4). The Mid-PD group needed more steps and a longer duration to perform the trials. They also showed a lower SD of VT and AP signals, lower harmonic ratios (VT, ML, and AP) and lower normalized peak power ML. In addition, the Mid-PD group showed higher asymmetry in step time, higher index of harmonicity (VT and AP) and higher width of peak power (VT and ML). The direction of these differences is consistent with gait impairment, except for stride regularity VT and normalized peak power VT. As a *post hoc* analysis, we removed corrections for confounders in the analyses of these variables, which led to lower and non-significant group differences. This suggests an over-compensation in the original results, mainly caused by gait speed. As for the Early-PD, the Mid-PD presented higher scores of gait-UPDRS III than the HC and the beta value for group effect was the largest.

### Confounders

Significant effects of gait speed were observed for most of the gait features in both comparative analyses, Early-PD vs. HC and Mid-PD vs. HC (Supplementary Appendix A, Tables A3, A4). Age affected eight comparisons between Early-PD and HC groups, and 11 comparisons between Mid-PD and HC groups. Medication status affected three comparisons between Early-PD and HC groups, and five between Mid-PD and HC groups. For gait-UPDRS III, only medication status significantly affected the Early-PD vs. HC comparison, whereas only age affected the Mid-PD vs. HC comparison. Age was the main confounder in the associations between gait features and gait-UPDRS III (Supplementary Appendix A, Table A5).

## Discussion

### Potential Progression Markers of Circular Gait in Early and Middle Stages of PD

The faster decline of gait features in Early-PD and Mid-PD groups relative to the HC confirms their potential as progression markers in these stages of the disease. For instance, a faster increase in number of steps, related to a decrease in stride duration, in Early-PD compared to HC, reflects impairments in step and stride regulation. This regulation is considered the central motor disruption of gait hypokinesia and requires an increase in cadence as a compensatory mechanism (Morris et al., 1994a,b, 1996). Aging lowers gait speed with consequent slower stepping (Menz et al., 2003b), however, the gait hypokinetic mechanism in PD involves a faster reduction in step length, reflected in our results (at least indirectly) as a faster decrease in stride times for the early-stage PD group compared to the HC. Moreover, in this study, the Early-PD group presented a faster reduction of harmonic ratios than the HC. These gait features are associated with impaired regulation of gait rhythmicity (Menz et al., 2003a), a motor impairment present in all stages of PD (Maetzler et al., 2009, 2012). The Mid-PD group showed a faster worsening of gait features related to variability and regularity (stride time variability, stride regularity) relative to the HC, reflecting progressive loss of gait consistency.

Our results, obtained from a linear walking task in the same cohort, suggested that turning and straight walking disentangle different features of gait that deteriorate in PD over time (Hobert et al., 2019). Another study investigating linear walking in PD found reductions in step length and swing time over a 18 months follow-up (Galna et al., 2015). However, out of sixteen proposed gait features, only these two gait features presented significant temporal changes. These observations suggest that straight gait may show different progression markers and may be less sensitive to progression in PD than circular gait.

The proposed progression markers were obtained with low-cost instrumentation and a limited measurement time. While short test durations limit the precision (Bruijn et al., 2009) and reliability (Lord et al., 2013) of the gait features studied and precluded including gait features that require longer times series (Bruijn et al., 2009), these results are in our view still important for implementation in clinical practice. The gait-UPDRS III scores reflected progression in the Early-PD with a larger change in this clinical rating than in any of the gait features. However, this was not the case for the Mid-PD. Based on the objective progression markers identified, worsening of gait in early PD seems to be mainly apparent in slowing of gait, coinciding with a reduced step time and increased gait asymmetry, as reflected in the harmonic ratios. These changes might be relatively well observable by the clinician. Thus, combining them with other observable changes not considered in our sensor-based assessment, such as a decreased arm swing, might have reflected in larger changes in the UPRDS III score. In Mid-PD, worsening of gait appears mainly reflected in decreased stride regularity. Such changes would be less detectable by visual observation, possibly explaining why objective gait features were more sensitive to change than the UPDRS III score. Still, the associations of the most promising progression markers of the gait turning paradigm with the respective gait-UPDRS scores indicate substantial construct validity of the former.

PD is a heterogeneous disease (Foltynie et al., 2002) with a variable expression and clinical heterogeneity in symptom severity, progression rate and disease profile (Postuma et al., 2016). The smaller number of progression markers in middle stages compared to early stages PD, may partly be explained by the decreasing rate of changes of motor symptoms with advancing disease (Poewe, 2009; Reinoso et al., 2015), compared to the steeper or even exponential progression of these symptoms in early stages (Nandhagopal et al., 2009). Interestingly, another study based on the MODEP data set investigated the potential of pegboard performance measures to assess progressive fine-motor dysfunction, and did only find relevant changes in the Early-PD group (Heinzel et al., 2017). Another reason for the relatively low number of continuously progressing gait parameters found in this study may be the study design *per se*. Clinical ratings indicate that relatively stable periods and even periods of improvement in symptoms may occur in PD (Reinoso et al., 2015).

### Baseline Differences Between Healthy Controls and Patients in Early and Middle Stages of PD

The number of significantly different features compared to the HC and their model coefficients were larger for the Mid-PD than for the Early-PD group. This observation is expected and consistent with the presence of more pronounced motor symptoms in middle stages than in early stages of PD (Maetzler et al., 2009). The presence of irregular gait patterns during turning in both groups of patients is in agreement with previous observations. For instance, a reduction in harmonic ratios, which is associated with degradation of walking stability, rhythmicity and control of the trunk during walking, has been observed in patients with PD who did not present step duration differences (Palmerini et al., 2013). This might be explained by the presence of bradykinesia, akinesia or axial and lower limb rigidity (Visser et al., 2007), which compromise trunk movement adaptations even at early stages of PD (Lowry et al., 2009). However, in this previous study, participants performed a turn of smaller angle and shorter duration than the turns assessed in our current study (Lowry et al., 2009), which might lead to different turning strategies.

Compromised control of dynamic stability during turning, as observed in both PD groups, increases the risk of falling (Mellone et al., 2016) and is likely reflected by longer test durations, a larger number of steps (Yang et al., 2016) (only observed in the Mid-PD) and reduced stride and step regularities (Rispens et al., 2014b). In similar vein, lower normalized peak powers of AP accelerations in the Early-PD and of ML accelerations in the Mid-PD potentially reflect lack of periodicity in their walking patterns (Weiss et al., 2011). This finding is consistent with larger width of peak power observed in the comparison of each PD group with the HC (Weiss et al., 2011). Moreover, diminished SD of trunk accelerations, as observed in the Mid-PD, has been shown to be associated with fall risk, probably reflecting a more conservative and less vigorous/intense walking pattern (Rispens et al., 2014b). In line with previous work in PD (Lamoth et al., 2002), we observed relatively large differences in indices of harmonicity between the HC and both PD groups, which may also indicate a less vigorous gait pattern in PD. Gait-UPDRS III scores presented a clear differentiation between both PD groups and the HC, although these scores might be biased by the subjectivity (e.g., expectations, prior experience) of the clinical rater, and their accuracy is critical (Espay et al., 2016).

### Confounders

The identification, reporting and adjustment for confounders in the analyses reported here are critical to ensuring that any of the observed changes in gait features can be confidently attributed to the progression of PD (Hubble et al., 2015). In this regard, accounting for gait speed is relevant, as performed in this study. Consistent with other studies, most features were speed dependent, including harmonic ratios (Menz et al., 2003a; Lowry et al., 2009), spectral content of accelerometry (Brach et al., 2010), gait variability (Brunelli et al., 2014), index of harmonicity (gait smoothness) (Lamoth et al., 2002) and magnitude-related measures (e.g., SD of signals or gait intensity; Menz et al., 2003a). Age (Imms and Edholm, 1981; Helbostad and Moe-Nilssen, 2003), and medication status effects (Blin et al., 1991) have also been reported in the literature as potential modifiers of gait patterns. However, in this study, a limited number of features required accounting for age and medication state, possibly due to adequate age-matching between groups, and a small percentage of data obtained ON medication, respectively. In the study of associations between gait features and clinical scores, age was identified as the most common confounder, probably associated with the inclusion of both Early-PD and Mid-PD groups together in a single PD group.

### Limitations

The statistical method employed in this analysis (GEE) does not account for exponential rates of progression, although the progression of neurodegeneration might be very rapid and non-linear, in particular at an early stage of PD (Heinzel et al., 2016). However, visual inspection of our data did not show exponential changes of the proposed gait features over the follow-up period. Given the length of the follow-up and the stratification into early-stage and middle-stage PD, we consider that linear models reasonably reflect the time course of progression.

A major strength of this study is its longitudinal design, which allowed identifying markers of progression. However, as more severely affected participants have higher attrition rates than their peers with better health status, simply due to logistic reasons (approaching the hospital, etc.), the study may have underestimated the overall rate of progression. We therefore performed a *post hoc* analysis with data that did not contain missing longitudinal data in annual intervals over five years. The results (Supplementary Appendix B, Table B1) were similar to the main findings. The inclusion of subjects who were only present at measurements in the first years may have limited the overall significance of the Time × Group interaction effect, although GEE analysis is expected to statistically overcome this limitation (Twisk, 2013) and including these data increased statistical power. In addition, variance caused by day-to-day differences or visit specific measurement errors was reduced by averaging biannual assessments. Results based on a *post hoc* analysis of data without averaging biannual measurements showed similar patterns (Supplementary Appendix B, Table B2). Overall, given the frequent assessments over a relatively long observation period and consideration of potential confounders, we believe that progression, limited to the span of this study, was investigated with sufficient detail.

We averaged data from both trials (in clock-wise and counter-clock-wise direction) without including the analysis of differences in gait features between these trials. The comparison between clock-wise gait features and counter-clock-wise features might lead to the detection of impairments related to the lateralization of PD, and therefore, it might reveal increasing gait asymmetry. Therefore, we evaluated gait feature differences between clock-wise and counter-clock-wise gait in a *post hoc* analysis (Supplementary Appendix B, Table B3). Except for asymmetry of step time, which increased faster in the Early-PD compared to the HC, and presented a trend (*p* = 0.06) toward higher values in the Mid-PD than in the HC at baseline, we did not find additional progression markers, probably due to variability in the lateralization of PD. We additionally performed two analyses (Supplementary Appendix B, Tables B4, B5) in which we evaluated gait features selected from specific protocols. In Table B4, the protocols were selected such that the most impaired side of patients with PD was on the inside of the circle; while in Table B5, the most impaired side was on the outside of the circle. Although in the same direction, these new results presented less gait features with significant Group × Time interactions, which indicates that the selection of gait features based on pathological lateral impairment does not add value to the identification of progression markers in PD.

In this study, we did not distinguish patients with the postural instability gait difficulty (PIGD) clinical phenotype, which have been suggested to show a more variable and rapid decline than patients with the tremor dominant (TD) clinical phenotype of PD (Jankovic, 2008). However, based on our clinical data, we classified all patients with PD of this study in the two clinical profiles (Stebbins et al., 2013): PIGD and TD, and compared each group with the HC and among them. None of the analyses led to any consistent progression difference. This indicates that the progression markers we identified are potentially useful across both patient groups.

In addition to clinical phenotypes, medication, specifically the levodopa equivalent daily dose, may affect disease progression and thus the rate of changes in the markers studied here. Unfortunately, the available data did not allow us to test for such an effect and any effect thereof is thus unaccounted for in our models. In addition, medication status at the time of the measurements varied between participants and measurement points, with 16.4% of measurements performed ON medication. Differences in medication state, combined with the presence of symptoms like dyskinesia and freezing of gait in some participants, may have affected outcomes. Although we controlled for confounding by medication status at the time of testing, this should be considered a limitation of this study.

The cut-off value used for “years after diagnosis” has as a disadvantage, since the follow-up period exceeds this cut-off value. Thus, patients at early stages of PD “shift” into the Mid-stage PD group during the observation period. However, we considered adequate such stratification, given that an examiner needs to decide at the beginning of the patient’s treatment how disease progression (and, eventually, treatment response) should be assessed. Moreover, this cut-off value was optimal from a statistical point of view, leading to similar sized groups. Using exactly this stratification approach, we could show in a previous publication (Heinzel et al., 2017) that pegboard assessment is a useful tool for the study of progression in early stages of PD, but not in mid-stages of the disease. In the present study, we could show that instrumented circular walking is an interesting progression marker in both, early and mid-stages of PD. Outside of this manuscript, we did perform all analyses with a merged group (all PD patients within a group), and obtained a limited number of significant results. Harmonic ratio and normalized peak power, obtained from VT acceleration did show significance for the interaction Group × Time. However, the mentioned results were not included in this study because this would lead to an over-representation of information from middle disease durations and relative under-representation of shorter and longer disease durations. Moreover, since an analysis of the pooled data did not yield satisfactory results, possibly due to the non-linearity of changes and due to the high within group (PD group) variability, we argued in favor of stratification to address the study of PD progression from the available data.

Finally, since the predictive power of instrumented gait assessment over circular trajectory was not tested in this study (i.e., predicting disease progression at the individual level), the findings cannot be considered as evidence for markers, but rather as potential markers of disease progression.

### Implications and Future Directions

The aim of the present longitudinal study, based on a 5 years follow-up was to identify potential markers of disease progression in PD from the assessment of circular gait patterns with a single BFS mounted on the low-back. For early-stage and middle-stage PD, we identified, respectively, five and three potential markers of progression from a total of 24 gait features assessed. At baseline, nine and fifteen features were found to be different between HC and patients at early-stage and middle-stage PD. While the clinical assessment of gait-UPDRS III was more sensitive than objective gait features in early-stage PD, this was not the case in middle-stage PD.

To our knowledge, this is the first study reporting objective progression markers from circular gait assessments in PD. Quantitative assessments permit monitoring of individual variability in motor symptoms, minimize subjectivity, and increase sensitivity to progression in PD. The progression markers identified may facilitate determining the potential effect of interventions on PD progression. Still, validation of the clinical relevance of the proposed markers is needed. Finally, the combination of the proposed protocol with a dual-task activity might further challenge the motor control of the participants, possibly revealing even stronger/more overt markers of disease progression.

## Data Availability Statement

The datasets generated and/or analyzed during the current study are not publicly available due to ethical regulations (the ethical proposal does not include a free data sharing option, and data can only be used in the frame of collaboration projects with the Tübingen study team), but are available from the corresponding author on reasonable request.

## Author Contributions

EM-A developed the study and method, analyzed the results and drafted the manuscript. IK contributed to the development of the study and method, participated in the interpretation of the data, and helped to draft the manuscript. SH supported in the statistical analysis and in the drafting of the manuscript. SR supported the development of the method for gait features extraction in Matlab and reviewed the manuscript. TH and SN collected the data. RvL participated in the coordination of the study and provided ideas to draft the manuscript. DB designed and coordinated the complete MODEP study. WM conceived and coordinated the complete MODEP study, supported the analysis and reviewed the manuscript. JvD participated in the coordination of the study, in the development of the method, in the interpretation of data, and in the drafting of the manuscript. All authors read and approved the final manuscript.

## Conflict of Interest Statement

McRoberts B.V. Company was the manufacturer of the DynaPort Hybrid sensor that was used in this study. RvL was the founder and owner of McRoberts B.V. and was involved in the coordination of the study and in drafting the manuscript. The remaining authors declare that the research was conducted in the absence of any commercial or financial relationships that could be construed as a potential conflict of interest.

## Abbreviations

AP: Anterior-posterior direction
BFS: Body-fixed-sensor
GEE: generalized estimating equations
HC: Healthy control
ML: Medio-lateral direction
MMSE: Mini Mental State Examination
MODEP: Modeling epidemiological data to study PD progression
PD: Parkinson’s disease
PIGD: postural instability gait difficulty
PSD: Power spectral density
SD: Standard deviation
SE: standard error
TD: tremor dominant
UPDRS: Unified Parkinson’s disease rating scale
VT: Vertical direction
