# Supplementary material for: Potential Markers of Progression in Idiopathic Parkinson’s Disease Derived From Assessment of Circular Gait With a Single Body-Fixed-Sensor: A 5 Year Longitudinal Study
Source: Front Hum Neurosci. 2019 Feb 19;13:59. doi: 10.3389/fnhum.2019.00059 (PMC6389786; doi:10.3389/fnhum.2019.00059)
Supplement: Supplementary file 3 [file Data_Sheet_3.docx]

Appendix A – Tables caption

**Table A.1. Flowchart visits.** Biannual visits performed by each of the participants. Complete datasets of 5 consecutive year visits were not always available in the MODEP cohort (HC: 52.0%; Early-PD: 59.1%; Mid-PD: 40.7%). This was partially due to the fact that some participants were recruited later in the MODEP study and therefore have not (yet) completed 10 visits, thus these participants may not be considered as drop-outs.

**Table A.2. Mean and standard deviation values of all gait features.** Mean and standard deviation (SD) of all features being not log transformed, nor z-score normalized. The data is presented for the three groups (Early-PD, Mid-PD and HC). Abbreviations: VT, vertical acceleration; ML, medio-lateral acceleration; AP, anterior-posterior acceleration.

**Table A.3. Results of GEE analysis for the comparison Early-PD vs HC.** This includes the factors Time, Group and the interaction Time*Group. For each factor, the β-value, SE (standard error) and a p-value were reported. All β-values were based on transformed and normalized data (log transformation and z-score normalization). In addition, the percentage of annual change relative to the first year was separately included for each group; particularly for the features that showed significance in the interaction Time*Group. The percentage of annual change was based on non-transformed and non-normalized data. Significant confounders that were accounted for the analysis are indicated as follows: “a” for gait speed effect, “b” for age effect and “c” for ON/OFF medication state effect. Note that the gait feature “Total duration” was not corrected for gait speed, this is indicated with *. Abbreviations: SD, standard deviation; gait-UPDRS III, motor section of Unified Parkinson's disease rating scale concerning gait assessment; VT, vertical acceleration; ML, medio-lateral acceleration; AP, anterior-posterior acceleration.

***Table A.4. Results of GEE analysis for the comparison Mid-PD vs HC.*** *This includes the factors Time, Group and the interaction Time*Group. For each factor, the β-value, SE (standard error) and a p-value were reported. All β-values were based on transformed and normalized data (log transformation and z-score normalization). In addition, the percentage of annual change relative to the first year was separately included for each group; particularly for the features that showed significance in the interaction Time*Group. The percentage of annual change was based on non-transformed and non-normalized data. Significant confounders that were accounted for the analysis are indicated as follows: “a” for gait speed effect, “b” for age effect and “c” for ON/OFF medication state effect. Note that the gait feature “Total duration” was not corrected for gait speed, this is indicated with *. Abbreviations: SD, standard deviation; gait-UPDRS III, motor section of Unified Parkinson's disease rating scale concerning gait assessment; VT, vertical acceleration; ML, medio-lateral acceleration; AP, anterior-posterior acceleration.*

***Table A.5. Results of GEE analysis for the association of gait features with clinical data (only for participants with PD).*** *This includes the single factor “Effect”, which was the clinical data of interest: Hoehn and Yahr score and the gait score of the UPDRS III test. For each factor, the β-value, SE (standard error) and a p-value were reported. All β-values were based on transformed and normalized data (log transformation, z-score interaction). Significant confounders which were accounted for the analysis are indicated as follows: “a” for gait speed effect, “b” for age effect and “c” for ON/OFF medication state effect. Note that the gait feature “Total duration” was not corrected for gait speed, this is indicated with *. Abbreviations: SD, standard deviation; gait-UPDRS III, motor section of Unified Parkinson's disease rating scale concerning gait assessment; VT, vertical acceleration; ML, medio-lateral acceleration; AP, anterior-posterior acceleration.*

Appendix B – Tables caption

**Table B.1. Results of GEE analysis for the comparison Early-PD vs HC and Mid-PD vs HC of features calculated from data that did not contain missing longitudinal data in year-intervals over 5 years.** This includes the factors Time, Group and the interaction Time*Group. For each factor, the β-value, SE (standard error) and a p-value were reported. All β-values were based on transformed and normalized data (log transformation and z-score normalization). In addition, the percentage of annual change relative to the first year was separately included for each group; particularly for the features that showed significance in the interaction Time*Group. The percentage of annual change was based on non-transformed and non-normalized data. Significant confounders that were accounted for the analysis are indicated as follows: “a” for gait speed effect, “b” for age effect and “c” for ON/OFF medication state effect. Note that the gait feature “Total duration” was not corrected for gait speed, this is indicated with *. Abbreviations: SD, standard deviation; gait-UPDRS III, motor section of Unified Parkinson's disease rating scale concerning gait assessment; VT, vertical acceleration; ML, medio-lateral acceleration; AP, anterior-posterior acceleration.

***Table B.2. Results of GEE analysis for the comparison Early-PD vs HC and Mid-PD vs HC of gait of features calculated from biannual visits data (i.e. extracted from each visit of the participants).*** *This includes the factors Time, Group and the interaction Time*Group. For each factor, the β-value, SE (standard error) and a p-value were reported. All β-values were based on transformed and normalized data (log transformation and z-score normalization). In addition, the percentage of annual change relative to the first year was separately included for each group; particularly for the features that showed significance in the interaction Time*Group. The percentage of annual change was based on non-transformed and non-normalized data. Significant confounders that were accounted for the analysis are indicated as follows: “a” for gait speed effect, “b” for age effect and “c” for ON/OFF medication state effect. Note that the gait feature “Total duration” was not corrected for gait speed, this is indicated with *. Abbreviations: SD, standard deviation; gait-UPDRS III, motor section of Unified Parkinson's disease rating scale concerning gait assessment; VT, vertical acceleration; ML, medio-lateral acceleration; AP, anterior-posterior acceleration.*

***Table B.3. Results of GEE analysis for the comparison Early-PD vs HC and Mid-PD vs HC of gait features absolute differences (between clock-wise and counter-clock-wise).*** *This includes the factors Time, Group and the interaction Time*Group. For each factor, the β-value, SE (standard error) and a p-value were reported. All β-values were based on transformed and normalized data (log transformation and z-score normalization). In addition, the percentage of annual change relative to the first year was separately included for each group; particularly for the features that showed significance in the interaction Time*Group. The percentage of annual change was based on non-transformed and non-normalized data. Significant confounders that were accounted for the analysis are indicated as follows: “a” for gait speed effect, “b” for age effect and “c” for ON/OFF medication state effect. Note that the gait feature “Total duration” was not corrected for gait speed, this is indicated with *. Abbreviations: SD, standard deviation; gait-UPDRS III, motor section of Unified Parkinson's disease rating scale concerning gait assessment; VT, vertical acceleration; ML, medio-lateral acceleration; AP, anterior-posterior acceleration.*

***Table B.4. Results of GEE analysis for the comparison Early-PD vs HC and Mid-PD vs HC of gait of features calculated from one of the protocols, CW or CCW. In the PD group, the selection is based on the main lateral impairment. Thus, main lateral impairment will be in the inner side of the circle. If right, the selected protocol was CW and opposite. In case of HC, the average of features from CW and CCW were included in the analysis.*** *This includes the factors Time, Group and the interaction Time*Group. For each factor, the β-value, SE (standard error) and a p-value were reported. All β-values were based on transformed and normalized data (log transformation and z-score normalization). In addition, the percentage of annual change relative to the first year was separately included for each group; particularly for the features that showed significance in the interaction Time*Group. The percentage of annual change was based on non-transformed and non-normalized data. Significant confounders that were accounted for the analysis are indicated as follows: “a” for gait speed effect, “b” for age effect and “c” for ON/OFF medication state effect. Note that the gait feature “Total duration” was not corrected for gait speed, this is indicated with *. Abbreviations: SD, standard deviation; gait-UPDRS III, motor section of Unified Parkinson's disease rating scale concerning gait assessment; VT, vertical acceleration; ML, medio-lateral acceleration; AP, anterior-posterior acceleration.*

***Table B.5. Results of GEE analysis for the comparison Early-PD vs HC and Mid-PD vs HC of gait of features calculated from one of the protocols, CW or CCW. In the PD group, the selection is based on the main lateral impairment. Thus, main lateral impairment will be in the outside part of the circle. If right, the selected protocol was CCW and opposite. In case of HC, the average of features from CW and CCW were included in the analysis.*** *This includes the factors Time, Group and the interaction Time*Group. For each factor, the β-value, SE (standard error) and a p-value were reported. All β-values were based on transformed and normalized data (log transformation and z-score normalization). In addition, the percentage of annual change relative to the first year was separately included for each group; particularly for the features that showed significance in the interaction Time*Group. The percentage of annual change was based on non-transformed and non-normalized data. Significant confounders that were accounted for the analysis are indicated as follows: “a” for gait speed effect, “b” for age effect and “c” for ON/OFF medication state effect. Note that the gait feature “Total duration” was not corrected for gait speed, this is indicated with *. Abbreviations: SD, standard deviation; gait-UPDRS III, motor section of Unified Parkinson's disease rating scale concerning gait assessment; VT, vertical acceleration; ML, medio-lateral acceleration; AP, anterior-posterior acceleration.*
